# Acute localized exanthematous pustulosis induced by paclitaxel^☆^

**DOI:** 10.1016/j.abd.2021.05.015

**Published:** 2022-04-02

**Authors:** Ana Carolina Tardin Rodrigues de Medeiros, Juliana Lopes Corrêa, Ademar Schultz Junior, Karina Demoner de Abreu Sarmenghi

**Affiliations:** Dermatology Outpatient Clinic, Hospital Santa Casa de Misericórdia de Vitória, Vitória, ES, Brazil

Dear Editor,

Acute localized exanthematous pustulosis (ALEP) is an atypical variant of acute generalized exanthematous pustulosis (AGEP), a rare drug reaction.^1^ It is an acute, localized subcorneal aseptic pustular eruption, usually affecting the face, cervical region, or thorax.^2^, ^3^ The pathophysiological mechanisms remain unclear, although 90% of ALEP cases are drug-induced.^1^ This case report describes a paclitaxel-induced ALEP case in a patient undergoing breast cancer treatment.

A 63-year-old woman complained of the appearance of pustules on an erythematous base, symmetrically distributed on the face (Fig. 1) one week after paclitaxel infusion used as neoadjuvant treatment for breast cancer. She reported pruritus and a local burning sensation and denied having a fever. No mucous membrane lesions were observed. The laboratory tests showed normal values ​​for leukocytes (8,500 mm^3^) and serum C-reactive protein. The pustule secretion culture was negative. The skin biopsy revealed intraepidermal subcorneal pustules and a superficial perivascular lymphocytic infiltrate (Fig. 2). Based on the clinical presentation and histopathological findings, the diagnosis of ALEP was made. The patient was treated with 40 mg of prednisone per day, and chemotherapy was discontinued, with complete remission of the lesions after 14 days (Fig. 3). The follow-up continued by slowly decreasing the dose of corticosteroids.

**Figure 1:**
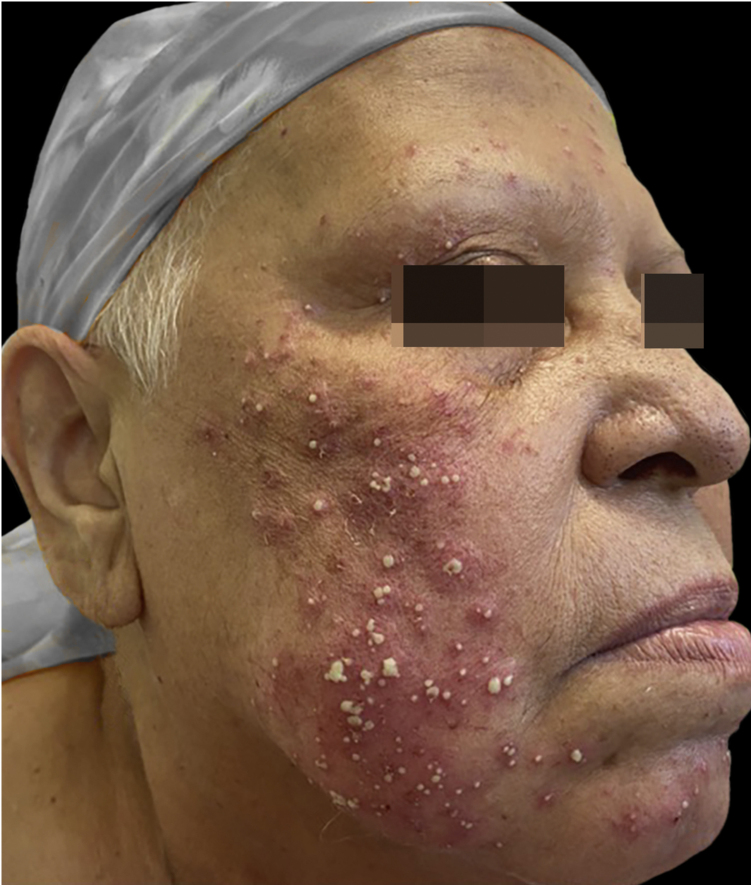
Presence of pustules on an erythematous base, symmetrically distributed on the face.

**Figure 2:**
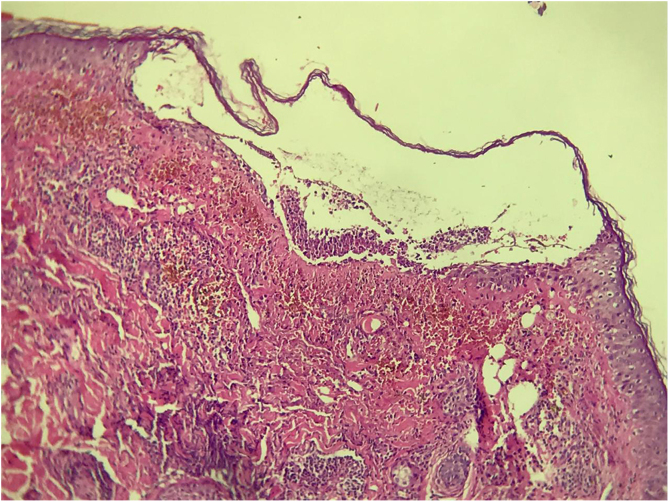
Histopathological examination stained with Hematoxylin & eosin, showing an intraepidermal subcorneal pustule, with superficial perivascular lymphocytic infiltrate, (Hematoxylin & eosin, ×10).

**Figure 3:**
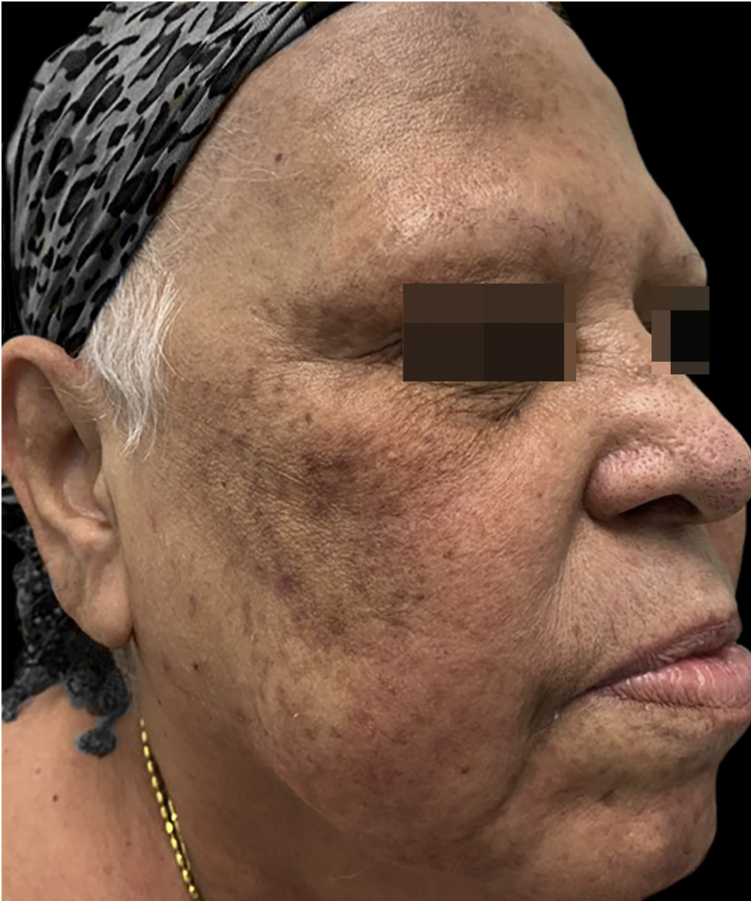
Post-inflammatory hyperchromia without the presence of pustular lesions.

Acute localized exanthematous pustulosis (ALEP) is a particular form of AGEP. Approximately 25 reports have been published in the literature, confirming that it is a rare drug reaction.^3^ Initially, the condition presents with sterile pustules located on the face, cervical region, or thorax after exposure to the drug, with spontaneous remission after discontinuation of the causative drug.^2^, ^3^ Fever and leukocytosis may be present, accompanied by pruritus or a burning sensation.^3^ About 90% of cases are drug-induced, with antibiotics, especially beta-lactams and macrolides, being the most common causative drugs.^1^, ^3^ Associations with docetaxel, a chemotherapeutic drug of the taxane class, have also been reported in two patients with breast cancer.^4^, ^5^

As ALEP is a self-limited disease, the mainstay of treatment is the withdrawal of the suspected drug, promoting symptom improvement within a few days. Support therapy with topical or oral corticosteroids may be appropriate for the treatment of pruritus and inflammation in prolonged cases.^3^

In conclusion, ALEP is an unusual drug-induced skin reaction, and the majority of published cases were secondary to the use of antibiotics. However, it is important to emphasize that chemotherapeutic agents of the taxane class may also be responsible for this skin reaction.

## Financial support

None declared.

## Authors’ contributions

Ana Carolina Tardin Rodrigues de Medeiros: Statistical analysis; design and planning of the study; drafting and editing of the manuscript; intellectual participation in the propaedeutic and therapeutic conduct of the studied cases; critical review of the literature.

Juliana Lopes Correa: Collection, analysis, and interpretation of data; drafting and editing of the manuscript; critical review of the literature.

Ademar Schultz Junior: Collection, analysis, and interpretation of data; design and planning of the study; drafting and editing of the manuscript.

Karina Demoner de Abreu Sarmenghi: Approval of the final version of the manuscript; effective participation in research orientation; intellectual participation in the propaedeutic and therapeutic conduct of the studied cases; critical review of the manuscript.

## Conflicts of interest

None declared.

## References

[1] Torrijos E.G., Calle M.P.C., Díaz Y.M., Lozano L.M., Ortega A.M., Bonilla P.A.G. (2017). Acute localized exanthematous pustulosis due to bemiparin. J Investig Allergol Clin Immunol.

[2] Prange B., Marini A., Kalke A., Hodzic-Avdagic N., Ruzicka T., Hengge U.R. (2005). Acute localized exanthematous pustulosis (ALEP). J Dtsch Dermatol Ges.

[3] Villani A., Baldo A., Salvatores G.F., Desiato V., Ayala F., Donadio C. (2017). Acute localized exanthematous pustulosis (ALEP): review of literature with report of case caused by amoxicillin-clavulanic acid. Dermatol Ther (Heidelb).

[4] Kim S.W., Lee U.H., Jang S.J., Park H.S., Kang Y.S. (2010). Acute localized exanthematous pustulosis induced by docetaxel. J Am Acad Dermatol.

[5] Ji Y.Z., Geng L., Qu H.M., Zhou H.B., Xiao T., Chen H.D. (2011). Acute generalized exanthematous pustulosis induced by docetaxel. Int J Dermatol.

